# Control of breathing by interacting pontine and pulmonary feedback loops

**DOI:** 10.3389/fncir.2013.00016

**Published:** 2013-02-13

**Authors:** Yaroslav I. Molkov, Bartholomew J. Bacak, Thomas E. Dick, Ilya A. Rybak

**Affiliations:** ^1^Department of Neurobiology and Anatomy, Drexel University College of MedicinePhiladelphia, PA, USA; ^2^Department of Mathematical Sciences, Indiana University – Purdue UniversityIndianapolis, IN, USA; ^3^Departments of Medicine and Neurosciences, Case Western Reserve UniversityCleveland, OH, USA

**Keywords:** respiratory central pattern generator, brainstem, ventrolateral respiratory column, pre-Bötzinger complex, pontine-medullary interactions, pulmonary feedback, control of breathing, apneusis

## Abstract

The medullary respiratory network generates respiratory rhythm via sequential phase switching, which in turn is controlled by multiple feedbacks including those from the pons and nucleus tractus solitarii; the latter mediates pulmonary afferent feedback to the medullary circuits. It is hypothesized that both pontine and pulmonary feedback pathways operate via activation of medullary respiratory neurons that are critically involved in phase switching. Moreover, the pontine and pulmonary control loops interact, so that pulmonary afferents control the gain of pontine influence of the respiratory pattern. We used an established computational model of the respiratory network (Smith et al., 2007) and extended it by incorporating pontine circuits and pulmonary feedback. In the extended model, the pontine neurons receive phasic excitatory activation from, and provide feedback to, medullary respiratory neurons responsible for the onset and termination of inspiration. The model was used to study the effects of: (1) “vagotomy” (removal of pulmonary feedback), (2) suppression of pontine activity attenuating pontine feedback, and (3) these perturbations applied together on the respiratory pattern and durations of inspiration (*T*_*I*_) and expiration (*T*_*E*_). In our model: (a) the simulated vagotomy resulted in increases of both *T*_*I*_ and *T*_*E*_, (b) the suppression of pontine-medullary interactions led to the prolongation of *T*_*I*_ at relatively constant, but variable *T*_*E*_, and (c) these perturbations applied together resulted in “apneusis,” characterized by a significantly prolonged *T*_*I*_. The results of modeling were compared with, and provided a reasonable explanation for, multiple experimental data. The characteristic changes in *T*_*I*_ and *T*_*E*_ demonstrated with the model may represent characteristic changes in the balance between the pontine and pulmonary feedback control mechanisms that may reflect specific cardio-respiratory disorders and diseases.

## Introduction

The respiratory rhythm and motor pattern controlling breathing in mammals are generated by a respiratory central pattern generator (CPG) located in the lower brainstem (Cohen, 1979; Bianchi et al., 1995; Richter, 1996; Richter and Spyer, 2001). The pre-Bötzinger complex (pre-BötC), located within the ventrolateral respiratory column (VRC) in the medulla, contains mostly inspiratory neurons (Smith et al., 1991; Rekling and Feldman, 1998; Koshiya and Smith, 1999). The pre-BötC, interacting with the adjacent Bötzinger complex (BötC), containing mostly expiratory neurons (Cohen, 1979; Ezure, 1990; Jiang and Lipski, 1990; Bianchi et al., 1995; Tian et al., 1999; Ezure et al., 2003), represents a core of the respiratory CPG (Bianchi et al., 1995; Tian et al., 1999; Rybak et al., 2004, 2007, 2008, 2012; Smith et al., 2007, 2009; Rubin et al., 2009; Molkov et al., 2010, 2011). This core circuitry generates primary respiratory oscillations defined by the intrinsic biophysical properties of respiratory neurons, the architecture of network interactions within and between the pre-BötC and BötC, and the inputs and drives from other brainstem compartments, including the pons, retrotrapezoid nucleus (RTN), raphé, and nucleus tractus solitarii (NTS). It has been suggested (Rybak et al., 2007, 2008; Smith et al., 2007) that these external inputs and drives may have a specific spatial mapping onto respiratory neural populations within the pre-BötC/BötC core network, so that changes in these inputs or drives can alter the balance in excitation between key populations within the core network, thereby affecting their interactions and producing specific changes in the respiratory motor patterns observed under different conditions.

Most CPGs controlling rhythmic motor behaviors in invertebrates and vertebrates operate under control of multiple afferent feedbacks and often provide feedback to the sources of their descending and afferent inputs hence allowing feedback regulation of the descending and afferent control signals (Dubuc and Grillner, 1989; Ezure and Tanaka, 1997; Blitz and Nusbaum, 2008; Buchanan and Einum, 2008), and this regulation often operates via presynaptic inhibition (Nushbaum et al., 1997; Ménard et al., 2002; Côté and Gossard, 2003; Blitz and Nusbaum, 2008).

As in other CPGs, afferent feedbacks are involved in the control of the mammalian respiratory CPG and the generation and shaping of the breathing pattern. Many peripheral mechano- and chemo-sensory afferents, including those from the lungs, tracheobronchial tree and carotid bifurcation, provide feedback signals involving in the homeodynamic control of breathing, cardiovascular function, and different types of motor behaviors coordinated with breathing, such as coughing (see Loewy and Spyer, 1990, for review). The NTS is the major integrative site of these afferent inputs. The present study focuses on the mechanoreceptor feedback mediated by pulmonary stretch receptors (PSRs). These mechanoreceptors respond to mechanical deformations of the lungs, trachea, and bronchi, and produce a burst of action potentials during each breath, thereby providing the central nervous system with feedback regarding rate and depth of breathing (see Kubin et al., 2006, for review). Activation of PSRs elicits reflex effects including inspiratory inhibition or expiratory facilitation (representing the so-called Hering-Breuer reflex), enhancement of early inspiratory effort, bronchodilatation, and tachycardia. PSR axons travel within the vagus nerve, and form excitatory synapses in NTS pump cells (Averill et al., 1984; Backman et al., 1984; Berger and Dick, 1987; Bajic et al., 1989; Anders et al., 1993; Kubin et al., 2006). Pharmacological microinjection and lesion studies (McCrimmon et al., 1987; Ezure et al., 1991, 1998; Ezure and Tanaka, 1996, 2004; Kubin et al., 2006) suggest that NTS pump cells mediate the Hering-Breuer reflex (lung-inflation induced termination of inspiration). Through pump cells, PSR-originating information alters the activity of CPG neurons in manners consistent with their proposed roles in rhythm generation.

The other feedback loop, important for the respiratory CPG operation, involves multiple pontine-medullary interactions. The pons (Kölliker-Fuse nucleus, parabrachial nucleus, A5 area, etc.) contains neurons expressing inspiratory (I)-, inspiratory-expiratory (IE)-, or expiratory (E)-modulated activity, especially in vagotomized animals (Bertrand and Hugelin, 1971; Feldman et al., 1976; Cohen, 1979; Bianchi and St. John, 1982; St. John, 1987, 1998; Shaw et al., 1989; Dick et al., 1994, 2008; Jodkowski et al., 1994; Song et al., 2006; Segers et al., 2008; Dutschmann and Dick, 2012). This modulation is probably based on reciprocal connections between medullary and pontine respiratory regions which were described in a series of morphological studies (Cohen, 1979; Bianchi and St. John, 1982; Nunez-Abades et al., 1993; Gaytan et al., 1997; Zheng et al., 1998; Ezure and Tanaka, 2006; Segers et al., 2008). The principal source of pontine influence on the medulla is thought to be the Kölliker-Fuse region in the dorsolateral pons, although other areas, including those from the ventrolateral pons, are also involved (Bianchi and St. John, 1982; Chamberlin and Saper, 1994, 1998; Dick et al., 1994; Fung and St. John, 1994a,b,c; Jodkowski et al., 1994, 1997; Morrison et al., 1994; St. John, 1998; Rybak et al., 2004; Dutschmann and Herbert, 2006; Mörschel and Dutschmann, 2009; Dutschmann and Dick, 2012). Pontine activity contributes to the regulation of phase duration as demonstrated by stimulation and lesion studies (Cohen et al., 1993; Jodkowski et al., 1994, 1997; Okazaki et al., 2002; Cohen and Shaw, 2004; Rybak et al., 2004; Dutschmann and Herbert, 2006; Mörschel and Dutschmann, 2009; Dutschmann and Dick, 2012). Stimulation of the Kölliker-Fuse or medial parabrachial nuclei induced a premature termination of inspiration (I-E transition) and extended expiratory phase. These effects were similar to the effects of vagal stimulation (Cohen, 1979; Hayashi et al., 1996). Also, the effects of both vagal and pontine stimulation appear to be mediated by the same medullary circuits that control onset and termination of inspiration (Haji et al., 1999; Okazaki et al., 2002; Rybak et al., 2004; Mörschel and Dutschmann, 2009; Dutschmann and Dick, 2012). Finally, the respiratory pattern in vagotomized animals with an intact pons is similar to that in animals without the pons and vagi intact. The above observations support the idea that the pontine nuclei mediate a function similar to that of the Hering-Breuer reflex.

Bilateral injections of NMDA antagonists (MK-801 and AP-5) into the rostral pons reversibly increase the duration of inspiration in vagotomized rats, and this increase is dose-dependent (Fung et al., 1994). This suggests that the rostral pons contains neurons with NMDA-receptors participating in the inspiratory off-switch mechanism. Morrison et al. (1994) showed that lesions of the parabrachial nuclei in the decerebrate, vagotomized, unanesthetized rat produced a significant (4-fold) increase in the duration of inspiration and a doubling of the duration of expiration, supporting a role for this pontine area in the regulation of the timing of the phases of respiration. This abnormal breathing pattern is known as apneusis. Administration of MK-801 into the rostral dorsolateral pons was shown to induce apneusis in vagotomized ground squirrels (Harris and Milsom, 2003). Systemic injection of MK-801 increases the inspiratory duration or results in an apneustic-like breathing in vagotomized and artificially ventilated rats (Foutz et al., 1989; Monteau et al., 1990; Connelly et al., 1992; Pierrefiche et al., 1992, 1998; Fung et al., 1994; Ling et al., 1994; Borday et al., 1998). Similarly, Jodkowski et al. (1994) showed that electrical and chemical lesions in the ventrolateral pons produced apneustic breathing in vagotomized rats. At the same time, apneustic breathing is not usually developed if the vagi remained intact and can be reversed by vagal stimulation, suggesting that NMDA receptors are not involved in the pulmonary (vagal) feedback mechanism.

Feldman et al. (1976) recorded cells in the rostral pons that exhibited respiratory modulation only when lung inflation, via a cycle-triggered pump, was stopped. The emergence of this respiratory-modulated activity suggests that afferent vagal input may have an inhibitory effect on the respiratory modulated cells in the pons (see also Feldman and Gautier, 1976; Cohen and Feldman, 1977). In the same work, it was noticed that this activity had no apparent influence on the tonic discharge of pontine neurons, suggesting that this inhibition might be presynaptic. Dick et al. (2008) recorded several hundred cells in the dorsolateral pons of decerebrate cats, artificially ventilated by a cycle-triggered pump before and after vagotomy. In their experiments, vagotomy led to either an emergence or facilitation of respiratory modulation in the pons. Sustained electrical stimulation of the vagus nerve elicited the classic Hering-Breuer reflex. Systemic or local blockade of NMDA receptors can result in an apneustic breathing pattern (Foutz et al., 1989; Connelly et al., 1992; Pierrefiche et al., 1992, 1998; Fung et al., 1994; Ling et al., 1994; Borday et al., 1998) similar to that demonstrated by pontine lesions or transections.

The specifics of feedback control in the brainstem respiratory CPG is that the latter operates under control of two control loops (pulmonary and pontine ones), which both regulate key neural interactions within the CPG, thereby affecting the respiratory rate, respiratory phase durations and breathing pattern, and, at the same time, interact with each other so that each of them may dominate in the control of breathing depending on the conditions and/or the state of the system. Such feedback interactions and a state-dependent feedback control of the CPG may have broader implication in other CPGs in vertebrates and/or invertebrates.

**Figure 1:**
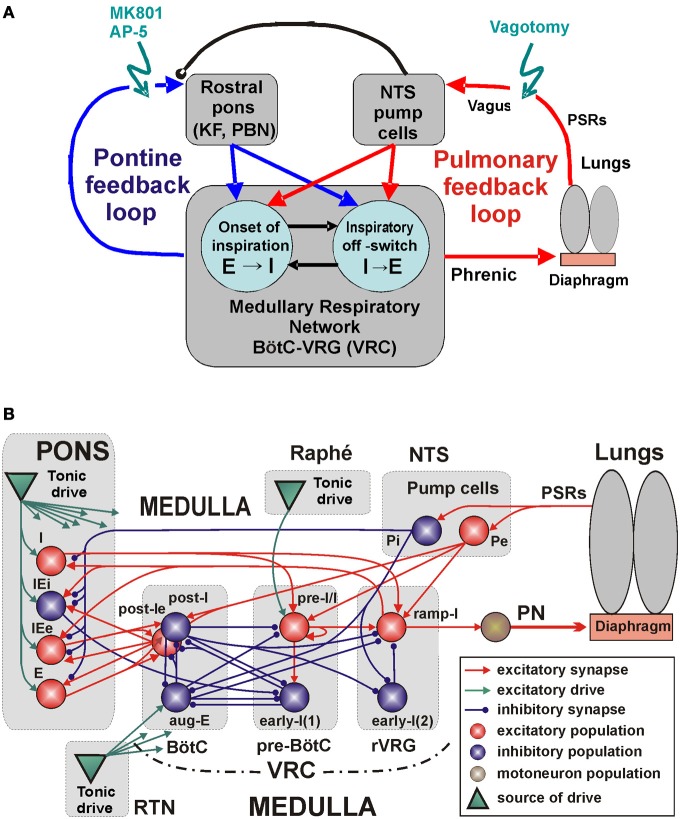
**The medullary respiratory network with pulmonary and pontine feedbacks. (A)** A general schematic diagram representing the respiratory network with two interacting feedback. See text for details. **(B)** The detailing model schematic showing interactions between different populations of respiratory neurons within major brainstem compartments involved in the control of breathing (pons, BötC, pre-BötC, and rVRG) and the organization of pulmonary and pontine feedbacks. Each neural population (shown as a sphere) consists of 50 single-compartment neurons described in the Hodgkin-Huxley style. The model includes 3 sources of tonic excitatory drive located in the pons, RTN, and raphé—all shown as green triangles. These drives, project to multiple neural populations in the model (green arrows; the particular connections to target populations are not shown for simplicity, but are specified in Table A3 in the Appendix). See text for details. *Abbreviations*: AP-5, amino-5-phosphonovaleric acid, NMDA receptor antagonist; BötC, Bötzinger complex; e, excitatory; E, expiratory or expiration; i, inhibitory; I, inspiratory or inspiration; IE, inspiratory-expiratory; KF, Kölliker-Fuse nucleus; MK801, dizocilpine maleate, NMDA receptor antagonist; NTS, Nucleus Tractus Solitarii; P, pump cells; PBN, ParaBrachial Nucleus; PN, Phrenic Nerve; pre-BötC, pre-Bötzinger Complex; PSRs, pulmonary stretch receptors; RTN, retrotrapezoid nucleus; r, rostral; VRC, ventral respiratory column; VRG, ventral respiratory group.

Specifically, our study focuses on the following major feedback loops involved in the control of breathing (Figure 1A): (1) the peripheral, pulmonary (vagal) loop that controls the medullary rhythm-generating kernel via afferent inputs from PSRs mediated by the NTS circuits, and (2) the pontine control loop, that provides pontine control of the respiratory rhythm and pattern. Our central hypothesis is that both the peripheral afferent and pontine-medullary loops control the respiratory frequency and phase durations via key medullary circuits responsible for the respiratory phase transitions (onset of inspiration, E-I, and inspiratory off-switch, I-E, see Figure 1A). In addition, these loops interact changing, balancing, and adjusting their control gain via interaction between NTS and VRC and pontine circuits. To investigate the involvement and potential roles of these feedback loops and their interactions with the medullary respiratory circuits we simulated the effects of suppression/elimination of each and both these feedbacks on the respiratory pattern and respiratory phase durations. The results of simulations were compared with the related experimental data and showed good qualitative correspondence hence providing important insights into feedback control of breathing.

## Methods

### Simulation package

All simulations in this study were performed using a neural simulation package NSM-3.0 developed at Drexel by Drs. Markin, Shevtsova, and Rybak and ported to the high-performance computer cluster systems running OpenMPI by Dr. Molkov. This simulation environment has been specifically developed and used for multiscale modeling and computational analysis of cross-level integration of: (a) the intrinsic biophysical properties of single respiratory neurons (at the level of ionic channel kinetics, dynamics of ion concentrations, synaptic processes, etc.); (b) population properties (synaptic interactions between neurons within and between populations with random distributions of neuronal parameters); (c) network properties (connectivity strength and type of synaptic interactions, with user-defined or random distribution of connections), (d) morpho-physiological structure (organization of interacting modules/compartments) (see Rybak et al., 2003, 2004, 2007, 2012; Smith et al., 2007; Baekey et al., 2010; Molkov et al., 2010, 2011). NSM-3.0 has special tools for simulation of various *in vivo* and *in vitro* experimental approaches, including suppression of specific ionic channels or synaptic transmission systems, various lesions/transections, application of various pharmacological, electrical and other stimuli to particular neurons or neural populations, etc.

### Modeling basis: neuronal parameters and ionic channel kinetics

The model presented in this paper continues a previously published series of models of neural control of respiration (Rybak et al., 2004, 2007; Smith et al., 2007; Baekey et al., 2010; Molkov et al., 2010, 2011) and, specifically, represents an extension of Smith et al. (2007) model. Following that model, each neuron type in the present model was represented by a population of 20–50 neurons. Each neuron was modeled as a single-compartment neuron described in the Hodgkin-Huxley (HH) style. These neuron models incorporated the currently available data on ionic channels in the medullary neurons and their characteristics. Specifically, the kinetic and voltage-gated and characteristics of fast (Na) and persistent (NaP) sodium channels in the respiratory brainstem were based on the studies of the isolated pre-BötC neurons in rats (Rybak et al., 2003). The kinetics and steady-state characteristics of activation and inactivation of high-voltage activated (CaL) calcium channels were based on the earlier studies performed *in vitro* (Elsen and Ramirez, 1998) and *in vivo* (Pierrefiche et al., 1999). Temporal characteristics of intracellular calcium kinetics in respiratory neurons were drawn from studies of Frermann et al. (1999). Other descriptions of channel kinetics were derived from previous models (Rybak et al., 2007; Smith et al., 2007).

Heterogeneity of neurons within each population was set by a random distribution of some neuronal parameters and initial conditions to produce physiological variations of baseline membrane potential levels, calcium concentrations, and channel conductances. A full description of the model and its parameters can be found in the Appendix. All simulations were performed using the simulation package NSM 3.0 (see above). Differential equations were solved using the exponential Euler integration method with a step of 0.1 ms. We utilized the high-performance computational capabilities of the Biowulf Linux cluster at the National Institutes of Health, Bethesda, MD (http://biowulf.nih.gov).

## Model architecture and operation in normal conditions

The main objective of this study was to investigate the mechanisms underlying control of the mammalian breathing pattern that is generated in the respiratory CPG circuits in the medulla and modulated by two major feedback loops, one involving interactions of medullary respiratory circuits with the lungs, and the other resulting from interactions of these circuits with the pontine circuits contributing to control of breathing (Figure 1A). We used an explicit computational modeling approach and focused on investigating the anticipated changes in the motor output (activity of the phrenic nerve, PN), specifically the changes in the duration of the inspiratory and expiratory phases under conditions of removal or suppression of the above feedback interactions (Figure 1A). The full schematic of our model is shown in Figure 1B. While developing this model, we used as a basis and extended the well-known large-scale computational model of the brainstem respiratory network developed by Smith et al. (2007). This basic model focused on the interactions among respiratory neuron populations within the medullary VRC. Similar to that model, the medullary respiratory populations in the present model (see Figure 1B) include (right-to-left): a ramp-inspiratory (ramp-I) population of premotor bulbospinal inspiratory neurons and an inhibitory early-inspiratory [early-I(2)] population—both in the rostral ventral respiratory group (rVRG); a pre-inspiratory/inspiratory (pre-I/I) and an inhibitory early-inspiratory [early-I(1)] populations of the pre-BötC; and an inhibitory augmenting-expiratory (aug-E) and inhibitory (post-I) and excitatory (post-Ie) post-inspiratory populations in the BötC. As suggested in the previous modeling studies (Rybak et al., 2004, 2007; Smith et al., 2007), these populations interact within and between the pre-BötC and BötC compartments and form a core circuitry of the respiratory CPG. In addition, multiple inputs and drives from other brainstem components, including the pons, RTN, NTS, and raphé affect interactions within this core circuitry and regulate its dynamic behavior and the motor output expressed in the activity of phrenic nerve (PN).

**Figure 2:**
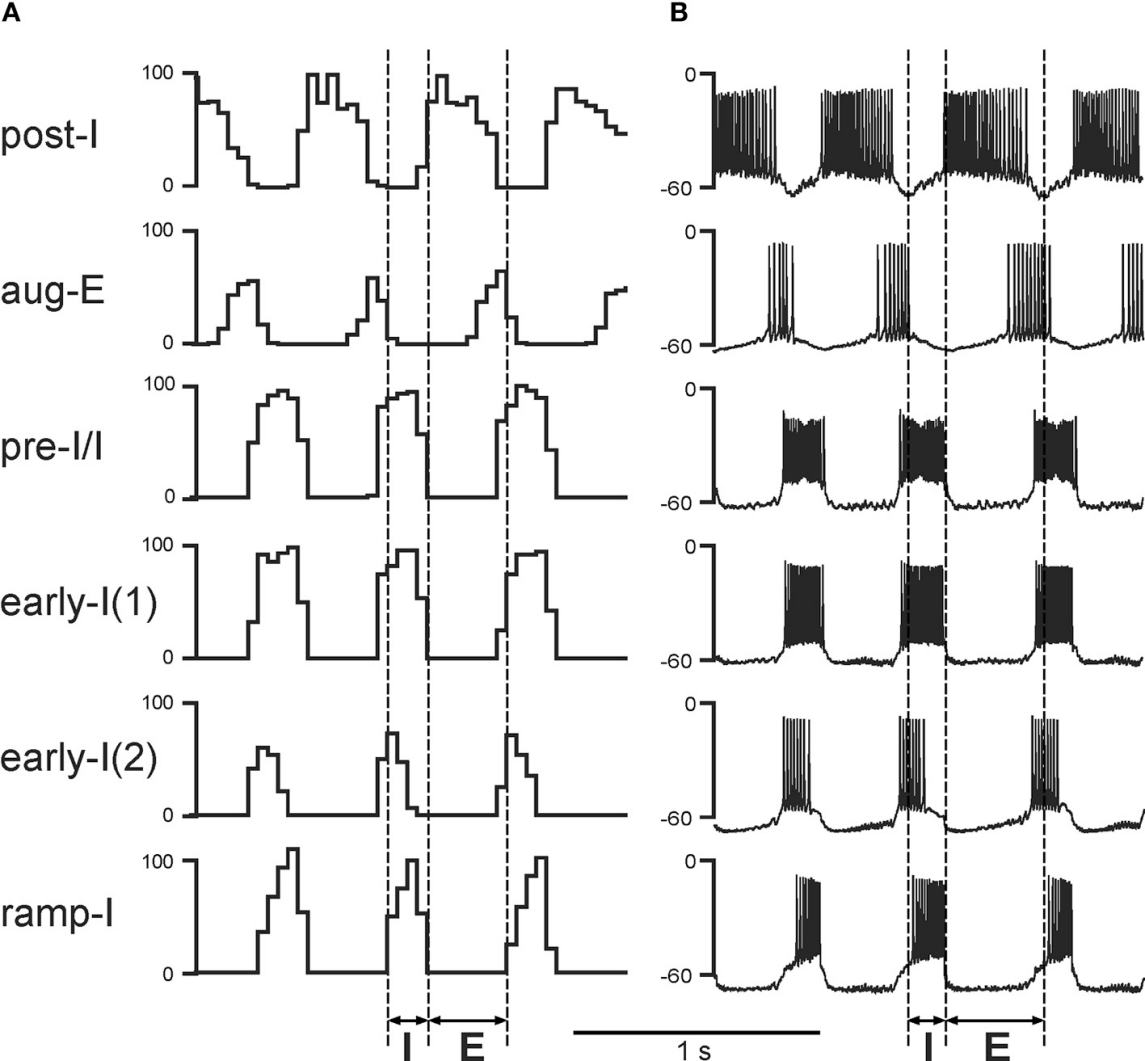
**Performance of the core medullary network under normal conditions (with both feedbacks intact). (A)** The activity of main neural populations of the core respiratory network under normal conditions. The shown population activities include (top–down): post-inspiratory (post-I) and augmenting expiratory (aug-E) (both in BötC); pre-inspiratory/ inspiratory (pre-I/I) and early-inspiratory [early-I(1)] (both in pre-BötC); early-inspiratory [early-I(2)] and ramp-inspiratory (ramp-I) (both in rVRG). The activity of each population is represented by the histogram of neuronal firing in the population (spikes/s; bin = 30 ms). **(B)** Traces of membrane potentials of the corresponding single neurons (randomly selected from each population). Vertical dashed line indicate the inspiratory (I) and expiratory (E) phases.

Respiratory oscillations in the basic and present models emerge within the BötC/pre-BötC core due to the dynamic interactions among: (1) the excitatory neural population, located in the pre-BötC and active during inspiration (pre-I/I); (2) the inhibitory population in the pre-BötC providing inspiratory inhibition within the network [early-I(1)]; and (3) the inhibitory populations in the BötC generating expiratory inhibition (post-I and aug-E). A full description of these interactions leading to the generation of the respiratory pattern can be found in previous publications (Rybak et al., 2004, 2007; Smith et al., 2007). Specifically, during expiration the activity of the inhibitory post-I neurons in BötC decreases because of their intrinsic adaptation properties (defined by the high-threshold calcium and calcium-dependent potassium currents) and augmenting inhibition from the aug-E neurons (Figures 1B and 2A,B). At some moment, the pre-I/I neurons of pre-BötC release from the deceasing post-I inhibition and start firing (Figure 2) providing excitation to the inhibitory early-I(1) population of pre-BötC and the premotor excitatory ramp-I populations of rVRG (Figure 1B). The early-I(1) population inhibits all post-inspiratory and expiratory activity in the BötC leading to the disinhibition of all inspiratory populations including the ramp-I hence completing the onset of inspiration (E-I transition). During inspiration early-I(1) inhibition of BötC expiratory neurons decreases due to intrinsic adaptation properties defined by the high-threshold calcium and calcium-dependent potassium currents (Figure 2). This decrease of inspiratory inhibition leads to the onset of expiration and termination of inspiration (inspiratory off-switch) (Figure 2). In the rVRG, the premotor ramp-I neurons receive excitation from the pre-I/I neurons and drive phrenic motoneurons and PN activity. The early-I(2) population shapes augmenting pattern of ramp-I neurons and PN. The PN projects to the diaphragm (Figure 1B) hence controlling changes in the lung volume (inflation/deflation) providing breathing.

**Figure 3:**
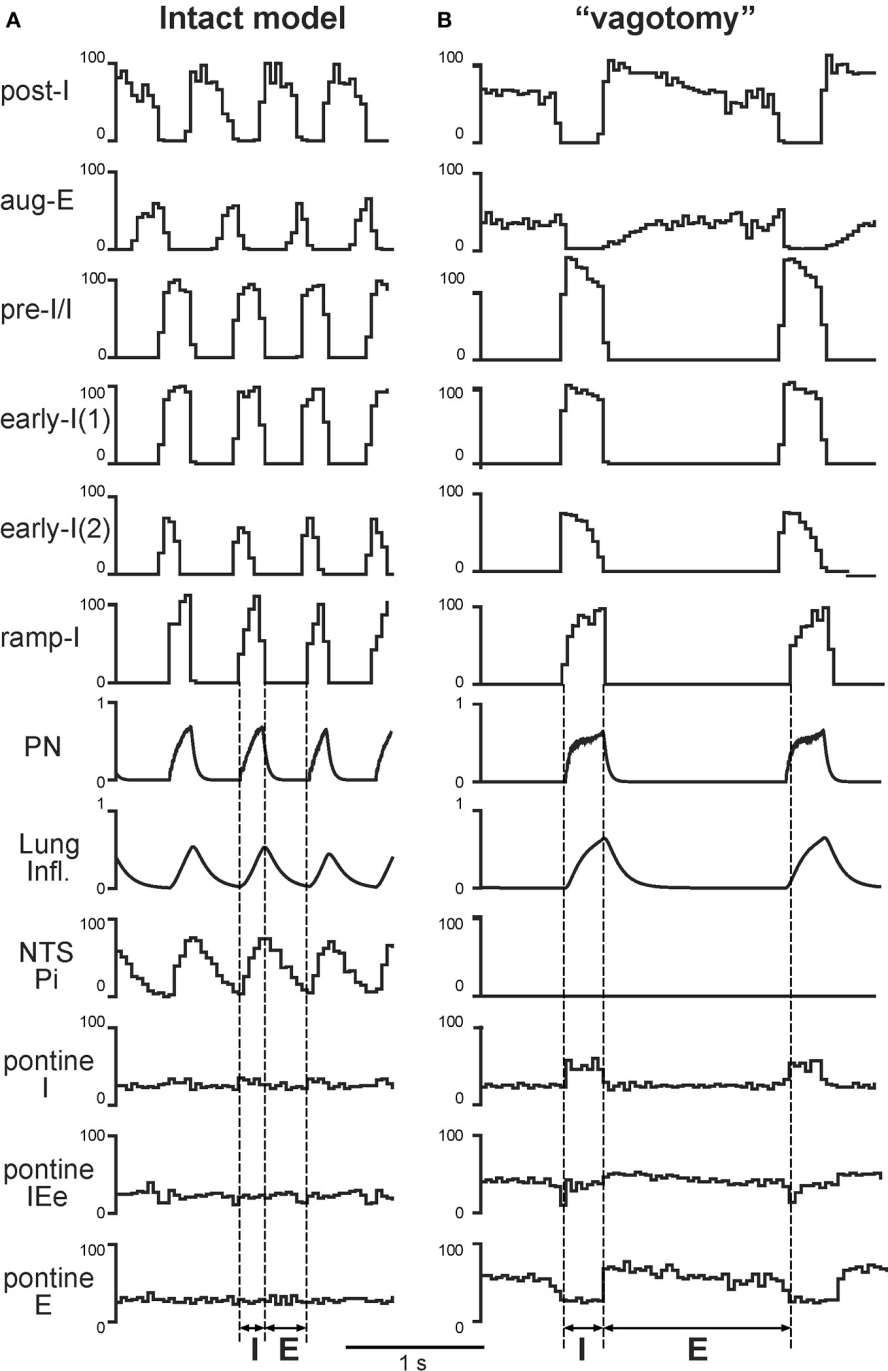
**Simulated vagotomy (removal of the pulmonary feedback).** Activity of major VRC (post-I, aug-E, early-I(1), pre-I/I, early-I(1), early-I(2), and ramp-I), NTS (Pi) and pontine (I, IEe, and E) neural populations, lung inflation and PN activity before **(A)** and after **(B)** simulated vagotomy. Vertical dashed line indicate the inspiratory (I) and expiratory (E) phases. See text for details.

The architecture of network interactions within the medullary VRC column (i.e., within and between the BötC, pre-BötC and rVRG compartments) in the present model is the same as in the preceding model of Smith et al. (2007). The extension of the basic model in the present study includes: (1) a more detailed simulation of the pontine compartment (in the Smith et al. model, the pontine compartment did not have neuron populations but simply provided tonic drive to medullary respiratory populations), (2) incorporation of suggested interactions between the pontine and medullary populations that form the pontine control loop in the model (Figures 1A,B), and (3) incorporation of the pulmonary (vagal) control loop that included models of the lungs and pump cells in the NTS (Figures 1A,B).

### Pontine feedback loop

As shown in multiple studies in cats and rats, many pontine neurons (including those in the Kölliker-Fuse and parabrachial nuclei) exhibit respiratory modulated activity, specifically with I-, IE-, E-, or EI-related activity (Bertrand and Hugelin, 1971; Feldman et al., 1976; Cohen, 1979; Bianchi and St. John, 1982; St. John, 1987, 1998; Shaw et al., 1989; Dick et al., 1994, 2008; Jodkowski et al., 1994; Song et al., 2006; Segers et al., 2008; Dutschmann and Dick, 2012). These neurons may have respiratory modulated activity summarized with background tonic firing or may express a pure phasic respiratory activity (especially in rats, e.g., see Ezure and Tanaka, 2006; Song et al., 2006). These pontine respiratory-modulated activities are probably based on specific axonal projections and synaptic inputs from the corresponding medullary respiratory neurons (Cohen, 1979; Bianchi and St. John, 1982; Nunez-Abades et al., 1993; Gaytan et al., 1997; Zheng et al., 1998; Ezure and Tanaka, 2006; Segers et al., 2008). In turn, pontine neurons (including those in the Kölliker-Fuse and parabrachial nuclei) project back to the medullary respiratory neurons contributing to the control of the respiratory phase durations and phase switching (Okazaki et al., 2002; Cohen and Shaw, 2004; Rybak et al., 2004; Dutschmann and Herbert, 2006; Mörschel and Dutschmann, 2009; Dutschmann and Dick, 2012). These mutual interactions between pontine and medullary respiratory neurons form what we refer to as a pontine (or pontine-medullary) control loop.

To simulate the pontine feedback loop, we incorporated in the pontine compartment of the model the following populations (see Figure 1B): the excitatory populations of neurons with inspiratory-modulated (I), inspiratory-expiratory-modulated (IEe) and expiratory-modulated (E) activities, and the inhibitory population of neurons with an inspiratory-expiratory-modulated (IEi) activity. As described above, pontine neurons with such types of modulated activity were found in both rat and cat. However, the existing experimental data on intrapontine and pontine-medullary interactions are insufficient and do not provide exact information on the specific connections between these neuron types; they only suggest general ideas and principles for organization of these interactions, such as the possible reciprocal interconnections between the pontine and medullary neurons with similar respiratory-related patterns (see references in the previous paragraph) and the existence of pontine projections to key medullary neurons involved in the respiratory phase switching (such as post-I, see references above). Therefore in the model, respiratory modulation of neuronal activity in pontine populations was provided by excitatory inputs from the medullary respiratory neurons with the corresponding phases of activity within the respiratory cycle. Specifically, the inspiratory modulation activity in the pontine I population was provided by excitatory inputs from the medullary ramp-I population, the IE modulation in the pontine IEe and IEi populations resulted from excitatory inputs from the medullary ramp-I and post-Ie populations, and the expiratory-modulation in the pontine E population was provided by inputs from the medullary post-Ie population. In addition, to simulate the presence of neurons with respiratory modulated phasic and tonic activities, each of the above four population was split into two equal subpopulations with neurons having the same properties and neuronal connections, but differed by tonic drive, which was received only by tonically active subpopulations (not shown in Figure 1B).

In turn, the pontine feedback in the model included (see Figure 1B): (1) excitatory inputs from the pontine I neurons (from both tonic and phasic subpopulations) to the medullary pre-I/I and ramp-I populations; (2) excitatory inputs from the pontine IEe neurons (both tonic and phasic subpopulations) to the medullary post-I population; (3) inhibitory inputs from the pontine IEi neurons (again both subpopulations) to the medullary early-I(1) population; and (4) excitatory inputs from the pontine E neurons (both subpopulations) to the medullary post-I, post-Ie, and aug-E populations. These neuronal connections from pons to medulla (especially pontine inputs to the medullary post-I and pre-I/I populations) allowed the pontine feedback to control operation of the respiratory network in the BötC/pre-BötC core and specifically to control the durations of the respiratory phases and phase switching. Specifically, the connection weights in the model were tuned so that (a) the durations of inspiration (*T*_*I*_) and expiration (*T*_*E*_) in the model without vagal feedback would be within the corresponding physiological ranges for the vagotomized rat *in vivo* (*T*_*I*_ = 0.2–0.55 s and *T*_*E*_ = 0.8–1.7 s, e.g., see Monteau et al., 1990; Connelly et al., 1992) and (b) after full suppression or removal of the pons, the value of *T*_*I*_ would dramatically increase (3–4 times or more) to be consistent with apneusis (Jodkowski et al., 1994; Morrison et al., 1994; Fung and St. John, 1995; St. John, 1998).

### Pulmonary (vagal) feedback loop

The busting activity of phrenic motoneurons produces rhythmic inflation/deflation of the lungs, which in turn causes rhythmic activation of PSRs projecting back to the medullary respiratory network within the vagus nerve and hence providing pulmonary (vagal) feedback. The activity of pulmonary afferents in the medulla is relayed by the NTS pump (P) cells. To simulate pulmonary feedback loop, we incorporated simplified models of the lungs and PSRs, so that changes in the lung volume were driven by the activity of PN (see Figures 1A,B). The resultant lung inflation activates PSRs that projected back activating the excitatory (Pe) and inhibitory (Pi) pump cells populations in the NTS. The latter finally projected to the VRC and pons (Figure 1B). Hence in the model, both Pe and Pi populations were involved in the Hering-Breuer reflex preventing over-inflation of the lungs. Specifically (Figure 1B), the Pe population excited the post-I population, which was based on the previous experimental data that both lung inflation and electrical stimulation of the vagus nerve produced an additional activation of decrementing expiratory neurons (Hayashi et al., 1996). Following the previous model (Rybak et al., 2004) we suggested that vagal feedback inhibits the early-I(1) population (in this model, via the Pi population). Both these interactions produced a premature termination of inspiration with switching to expiration and a prolongation of expiration.

### Interactions between the loops

As mentioned in the section “Introduction,” the respiratory-modulated activity in the pons is usually much stronger in the absence of lung inflation and in vagotomized animals (e.g., see Feldman et al., 1976; Dick et al., 2008). One explanation for these effects is that the respiratory-modulated activity in the pons is suppressed by vagal afferents via NTS neurons projecting to the pons. There is indirect evidence that this suppression is based on presynaptic inhibition (Feldman and Gautier, 1976; Dick et al., 2008). Therefore in our model, this presynaptic inhibition is provided by the Pi population of NTS and affects all excitatory synaptic inputs from medullary to pontine neural populations (Figure 1B). Therefore, this presynaptic inhibition suppresses the respiratory modulation in the activities of pontine neurons and reduces the influence of pontine feedback on the medullary respiratory network operation and the respiratory pattern generated. Because of the lack of specific data, the synaptic weighs of connections from both pump cell populations (Pe and Pi) were set so that (a) significantly reduce the respiratory nodulation in all types of pontine neurons and (b) keep the durations of inspiration and expiration in simulations with vagal feedback intact within their physiological ranges for the rat *in vivo* (*T*_*I*_ = 0.17–0.3 s and *T*_*E*_ = 0.3–0.5 s, e.g., see Connelly et al., 1992).

### Simulation of vagotomy (pulmonary feedback removal)

**Figure 4:**
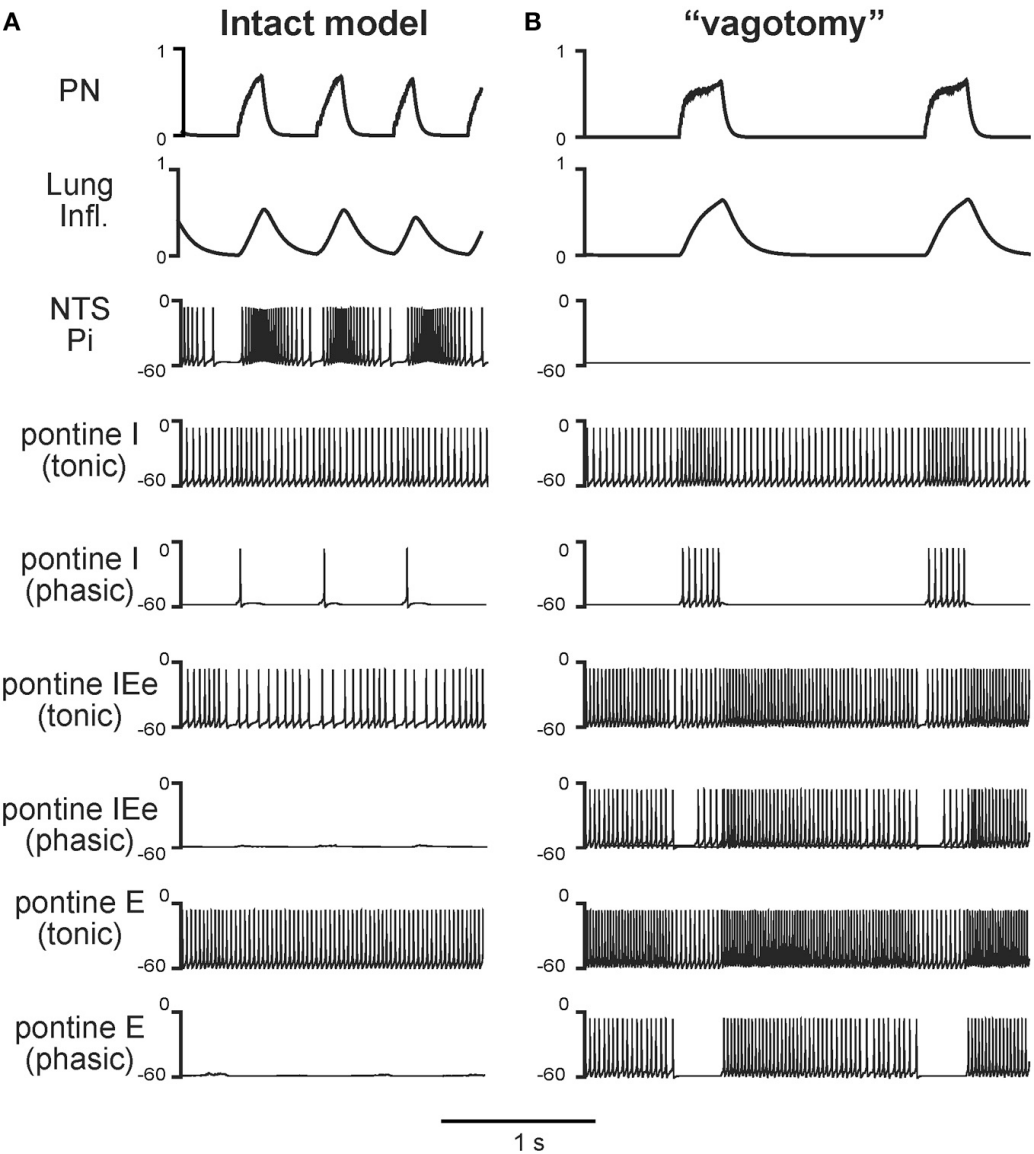
**Respiratory modulation in the activity of pontine neurones before (A) and after (B) simulated vagotomy.** The changes of phrenic activity (PN) and the lung inflation are shown at the top. Below these graphs, membrane potentials traces of representative single neurons from the Pi and pontine populations (tonic and phasic subpopulations) are shown. See text for details.

**Figure 5:**
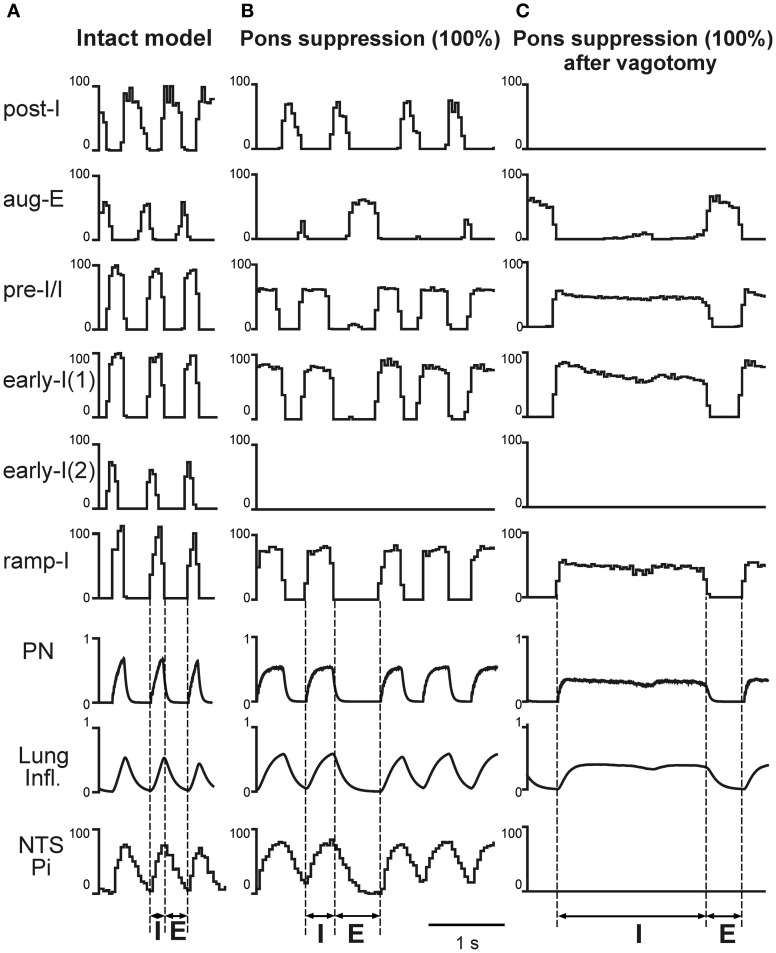
**The effects of pontine suppression before and after simulated vagotomy.** Activity of major medullary [post-I, aug-E, early-I(1), pre-I/I, early-I(1), early-I(2), and ramp-I], NTS (Pi) and pontine (I, IEe, and E) neural populations, lung inflation and PN activity under control conditions **(A)** and following the 100% suppression of pontine activity before **(B)** and after **(C)** simulated vagotomy. The activity pattern shown in **(C)** represents typical apneusis. Vertical dashed line indicate the inspiratory (I) and expiratory (E) phases. See text for details.

Under normal conditions the “intact” model generated the respiratory pattern with the duration of inspiration *T*_*I*_ = 0.189 ± 0.046 s and the duration of expiration *T*_*E*_ = 0.388 ± 0.064 s (Figures 2, 3A, 4A, and 5A). “Vagotomy” was simulated by breaking the pulmonary feedback, specifically by a removal of afferent inputs from PSRs to the pump cells in the NTS (Figure 1A). The resultant changes in the activity of different neural populations and in the output respiratory pattern in the model after simulated vagotomy are shown in Figures 3B and 4B. As a result of vagotomy the pump cells (Pi and Pe populations) become silent (only the activity of Pi is shown in Figures 3B and 4B; the activity of Pe population is similar, i.e., it also becomes silent). This eliminates the excitatory effect of lung inflation (PSR) on the post-I population (and post-Ie, pre-I/I, and ramp-I), mediated by Pe, and its inhibitory effect on the aug-E population, provided by Pi (Figure 1B). This also eliminates the pulmonary (vagal) control of respiratory phase switching and phase durations. However, this breaking of the pulmonary feedback also removes the presynaptic inhibition of all medullary inputs to pontine neural populations (provided in the intact case by the NTS's Pi population) hence increasing respiratory-modulated activities in the pontine neurons involved in the feedback control of the respiratory network operation (Figures 1A,B). This therefore increases the gain of pontine feedback and its role in the control of respiratory phase switching and phase durations. Figure 3 shows that the vagotomy resulted in increases in the respiratory-modulated activity of pontine populations, a prolongation of inspiration (*T*_*I*_ = 0.277 ± 0.108 s), and a dramatic increase in the expiratory phase duration (*T*_*E*_ = 0.938 ± 0.065 s). Figure 4 shows that the applied vagotomy produced a significant increase of inspiratory (I), inspiratory-expiratory (IE), and expiratory (E) modulation in the activity of the corresponding pontine neurons with tonic activity and releases the corresponding firing in pontine neurons with phasic I, IE, and E activities not active in the intact case.

### Simulation of pontine feedback suppression with and without pulmonary feedback

**Figure 6:**
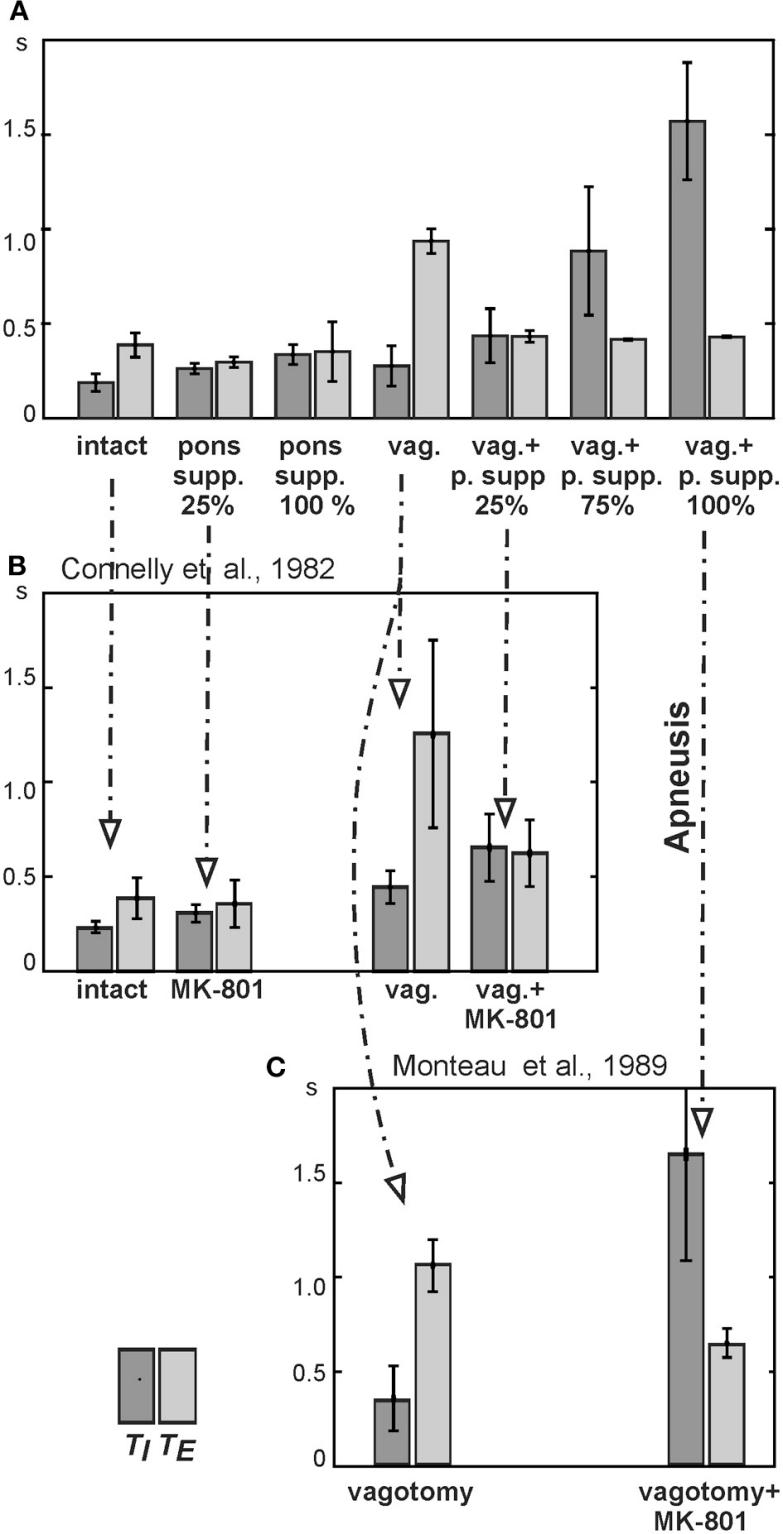
**Changes in the durations of inspiration (*T*_*I*_) and expiration (*T*_*E*_) following pontine suppression and/or vagotomy. (A)** Changes in *T*_*I*_ and *T*_*E*_ following the simulated pontine suppression at different degrees (25%, 75%, and 100%) before and after (vag. +) vagotomy. **(B)** Changes in *T*_*I*_ and *T*_*E*_ in the study of Connelly et al. (1992): diagrams are built for spontaneously breathing Wistar rats under control conditions and after administration of NMDA blocker MK-801 before and after vagotomy. **(C)** Changes in *T*_*I*_ and *T*_*E*_ in the study of Monteau et al. (1990) performed in anaesthetized vagotomized rats using MK-801 administration.

A complete removal of the pons (i.e., a removal of pontine feedback) in the model with an intact pulmonary feedback produced a prolongation of inspiration (*T*_*I*_ = 0.337 ± 0.052 s) and a slightly reduced in average (in comparison to the intact model) but highly variable expiratory duration (*T*_*E*_ = 0.353 ± 0.159 s) characterized by occasional deletions of aug-E bursts (see Figures 5B and 6A). To compare our simulations with the existing experimental data on the effects of pontine suppression by local injections of MK801, a blocker of NMDA receptors, that might not completely suppress the excitatory synaptic transmission in the pontine neurons and their activity, we also simulated a partial suppression of excitatory synaptic weights in the pontine compartment (e.g., by 25% see Figure 6A). Such partial suppression produced a visible prolongation of inspiration (*T*_*I*_ = 0.262 ± 0.028 s with *T*_*E*_ = 0.297 ± 0.028 s at 25% suppression, Figure 6A).

In contrast to pontine suppression with the intact pulmonary feedback, the same procedures after vagotomy led to a dramatic increase in the average duration of inspiration (making the inspiratory duration highly variable) at relatively constant duration of expiration (Figures 5C and 6A). This prolongation of inspiration after vagotomy increased with the degree of pontine suppression (reducing the weights of excitatory synaptic inputs to pontine neurons) (Figure 6A) and accompanied by a suppression or full elimination of post-I activity and reduced amplitude of integrated PN (Figure 5C). Both these features are typical for apneusis (see Cohen, 1979; Wang et al., 1993; Jodkowski et al., 1994; Morrison et al., 1994; Fung and St. John, 1995; St. John, 1998). The durations of inspiration and expiration after vagotomy at different degrees of pontine suppression were the following: *T*_*I*_ = 0.437 ± 0.143 s with *T*_*E*_ = 0.433 ± 0.030 s at 25% suppression; *T*_*I*_ = 0.885 ± 0.339 s with *T*_*E*_ = 0.417 ± 0.004 s at 75% suppression; and *T*_*I*_ = 571 ± 0.310 s with *T*_*E*_ = 0.431 ± 0.003 s at 100% suppression.

The results of our simulations reflecting changes in *T*_*I*_ and *T*_*E*_ following different combinations of vagotomy with pontine suppression at different degrees are shown together in Figure 6A. Our general conclusions made from these simulations are the following. (1) A suppression of pontine activity with the intact pulmonary feedback leads to a moderate prolongation of inspiration, slight shortening of expiration, and an increase in variability of *T*_*E*_ (with 100% pontine suppression). (2) The simulated vagotomy (with the intact pontine-medullary interactions) causes a moderate prolongation of inspiration with an increase in variability of *T*_*I*_ and a strong prolongation of expiration. (3) Combination of both perturbations does not produce visible effects on *T*_*E*_, but leads to a significant prolongation of inspiration (increasing with the degree of pontine suppression), increasing of *T*_*I*_ variability, and other typical characteristics of apneusis (suppressed post-I activity and reduced PN amplitude).

### Comparison with experimental data

**Figure 7:**
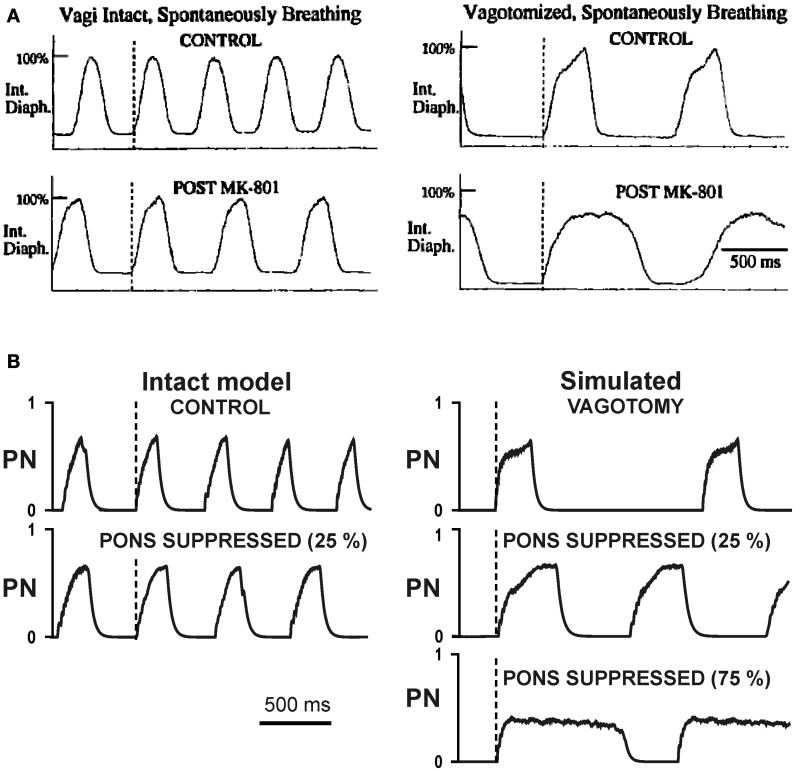
**Changes in the breathing pattern (phrenic activity, PN) following MK-801 application (pontine suppression in the model) before and after vagotomy. (A)** Changes in integrated phrenic nerve activity (Int. Diaph.) from spontaneously breathing Wistar rats before (top traces) and after (bottom traces) NMDA channel blockade, before (left diagrams) and after (right diagrams) vagotomy (from Connelly et al., 1992) **(B)** Changes in integrated phrenic nerve activity (PN) in our simulations before (top traces) and after (bottom traces) simulated pontine suppression, before (left diagrams) and after (right diagrams) simulated vagotomy.

To test our model, we performed simulation with 25%, 75%, and 100% suppression of the pontine control loop before and after simulated vagotomy (removal of the pulmonary feedback). The resultant changes in *T*_*I*_ and *T*_*E*_ are shown in Figure 6A. To compare these simulation results with the related experimental data, we built similar diagrams from the early study of Connelly et al. (1992), which examined spontaneously breathing in Wistar rats during the administration of NMDA blocker MK-801 before and after vagotomy (Figure 6B). In this study, the experiments on Wistar rats (in contrast to the Sprague-Dawley strain) did not end with apneusis, due to (in our opinion) an insufficient suppression of the pontine feedback by the performed MK-801 injections. Nevertheless, the effects of vagotomy and MK-801 administration on *T*_*I*_ and *T*_*E*_ before and after vagotomy reported in Connelly et al. study are qualitatively similar to our simulations with 25% suppression of pontine feedback (see Figures 6A,B). Specifically, the 25% pontine suppression in our simulations and the administration of MK-801 in Connelly et al. experiments result in an increase of *T*_*I*_ and slight reduction of *T*_*E*_ before vagotomy and in a significant prolongation of inspiration after vagotomy. In addition, vagotomy alone without other perturbations in both cases results in an increase of *T*_*I*_ and significant prolongation of *T*_*E*_ (see Figures 6A,B). Moreover, the changes in the respiratory frequency and the shape and amplitude of integrated phrenic activity after vagotomy and/or pontine suppression in our model are similar to that in the experimental studies with MK-801 administration (Figure 7). The other comparison of our simulations was made with the experimental study of Monteau et al. (1990) performed in anaesthetized vagotomized rats by using MK-801 administration, which results are summarized in Figure 6C. This study did demonstrate that MK-801 application after vagotomy produced switching from a normal breathing pattern to the typical apneusis. The relationships between *T*_*I*_ and *T*_*E*_ in our simulation after vagotomy and their changes following 100% pontine suppression (apneusis) are similar to these in the Monteau et al. study (see Figures 6A,C).

## Discussion

The results of our simulations promote the concept that both pulmonary and pontine feedback loops contribute to the control of the respiratory pattern and, specifically, the durations of inspiration (*T*_*I*_) and expiration (*T*_*E*_). Furthermore, our modeling results are consistent with the previous suggestion of specific interactions between these feedback loops, in particular that the PSR afferents involved in the pulmonary control of *T*_*I*_ and *T*_*E*_ attenuate the gain of the pontine control of these phase durations (via the presynaptic inhibition of excitatory inputs from medullary to pontine populations) (Feldman and Gautier, 1976; Feldman et al., 1976; Cohen and Feldman, 1977; Cohen, 1979; Mörschel and Dutschmann, 2009). Nevertheless, according to our simulations, pontine activity still plays a role in the control of inspiration and expiration even when the pulmonary feedback is intact, although the gain of this pontine control is significantly reduced by the presynaptic inhibition. This presynaptic inhibition is expected to suppress the respiratory modulation in the activity of pontine neurons expressing either tonic or phasic firing patterns (Feldman and Gautier, 1976; Feldman et al., 1976; Cohen and Feldman, 1977; Cohen, 1979; St. John, 1987, 1998; Shaw et al., 1989; Dick et al., 1994, 2008; Song et al., 2006; Segers et al., 2008), which is reproduced by our model (Figure 4). Also, the model offers a plausible mechanistic explanation for the previous experimental findings that injection of NMDA antagonists in the dorsolateral pons (specifically in the Kölliker-Fuse area) leads to a prolongation of inspiration and to apneusis in the case of a lack of pulmonary feedback (Foutz et al., 1989; Connelly et al., 1992; Pierrefiche et al., 1992, 1998; Fung et al., 1994; Ling et al., 1994; Bianchi et al., 1995; Borday et al., 1998; St. John, 1998).

In contrast to previous suggestions and models (Okazaki et al., 2002; Cohen and Shaw, 2004; Rybak et al., 2004; Dutschmann and Herbert, 2006; Mörschel and Dutschmann, 2009; Dutschmann and Dick, 2012), the mechanisms of action of the two feedbacks considered in the current model are not exactly symmetric. Excitatory inputs from both these feedbacks (from PSRs via the NTS's Pe cells, and from the pontine I, IEe, and E populations) activate the ramp-I, pre-I/I, post-Ie, and post-I medullary populations (see Figure 1B). The majority of these excitatory connections are the ones activating the inhibitory post-I population that controls the inspiratory off-switching, i.e., the timing of inspiratory phase termination and *T*_*I*_, and those activating the excitatory pre-I/I population which, in a balance with the inputs to post-I, control the onset of inspiration (and *T*_*E*_). However the effect of these excitatory inputs from the two feedbacks on the medullary circuitry is not identical and depends on the particular synaptic weights and the activity pattern of the inhibitory NTS's Pi cells providing presynaptic inhibition of medullary inputs to the pontine neurons (Figure 1B). The organization of inhibitory inputs of these feedbacks to the medullary populations in the model is different. While the pulmonary feedback inhibits the aug-E population (via PSRs and Pi cells) causing a complex effect on the respiratory pattern, the pontine IEi population inhibits the early-I(1) population hence promoting expiration, which is clearly seen after vagotomy (Figure 1B).

It is important to mention that the current model of the medullary core respiratory circuits in the VRC (including the BötC, pre-BötC, and rVRG) used in our model was derived from the model of Smith et al. (2007) without significant changes. Starting with that first publication, this basic model (with necessary additions) was able to reproduce multiple experimental results, including the characteristic changes of the respiratory pattern following a series of pontine and medullary transections and effect of riluzole (persistent sodium current blocker) on the intact and sequentially reduced *in situ* preparation (Rybak et al., 2007; Smith et al., 2007), the emergence of the additional late-expiratory oscillations in the RTN/parafacial respiratory group (RTN/pFRG) during hypercapnia and interactions between the BötC/pre-BötC and RTN/pFRG oscillators (Abdala et al., 2009; Molkov et al., 2010), the effects of baroreceptor stimulation and the respiratory-sympathetic coupling including this following the intermittent hypoxia (Baekey et al., 2010; Molkov et al., 2011; Rybak et al., 2012), etc. The extended model described here was also able to reproduce the above behaviors, including the biologically plausible changes of membrane potentials and firing patterns of different respiratory neurons (Figure 2B). The ability of the extended model to reproduce the experimentally observed effects of the two feedback loops provides an additional support for the model of the core respiratory circuits used in all these previous models.

The exact mechanisms of pontine control of breathing are not well-understood and the pontine-medullary connections incorporated in the model are currently speculative. However, the general importance of the pons in the control of the respiratory pattern is well-recognized (see Dutschmann and Dick, 2012, for review). Studies utilizing the classic neurophysiological approaches of lesioning, stimulating and recording neurons have established that the lateral pons influences not only phase duration, phrenic amplitude, and response to afferent stimulation, but also the dynamic changes in respiratory pattern associated with persistent stimuli. For instance, blocking neural activity in the dorsolateral pons not only prolongs inspiration but also blocks the adaptation to vagal stimulation (Siniaia et al., 2000), and the shortening of expiration associated with repeated lung inflation (Dutschmann et al., 2009). Thus, the pons is not only intimately involved in the initial response to various stimuli, but also in the complex processes of accommodation and habituation. In the cardiovascular control system, parabrachial stimulation attenuates the NTS response to carotid sinus nerve stimulation by inhibition of NTS neurons receiving these inputs (Felder and Mifflin, 1988).

With normally operating pontine-medullary interactions, the simulated vagotomy results in a prolongation of inspiration and significant increase of the expiratory duration (Figures 3B and 6A). However, despite these changes, the breathing pattern after vagotomy remains similar to that in eupnea (Figure 3). This maintenance of the eupneic breathing pattern occurs because the control performed by the pulmonary loop is now partly mimicked by the pontine loop, whose gain is increasing after vagotomy, as the latter removes the presynaptic inhibition of medullary inputs to pontine neurons (Figure 1B). Our model suggests that the pulmonary feedback yet performs the major function in the control of respiratory phase transitions and phase durations, and that a removal of this control loop places the full responsibility for this control on the pontine feedback loop.

The complementary role of the pontine and pulmonary feedbacks in control of phase duration (especially *T*_*I*_) in our model is consistent with the classical interpretation of their function in respiratory control (see Dutschmann and Dick, 2012, for review). In particular, a premature termination of inspiration and switching to expiration can be elicited by stimulation of either the rostral pons or the pulmonary afferents (Bertrand and Hugelin, 1971; Cohen, 1979; Oku and Dick, 1992; Wang et al., 1993; St. John, 1998; Haji et al., 1999; Okazaki et al., 2002; Rybak et al., 2004; Dutschmann and Herbert, 2006). This observation was explained by their common excitatory input on the post-inspiratory neurons in the medullary VRC which are critically involved in this phase transition (Okazaki et al., 2002; Rybak et al., 2004; Dutschmann and Herbert, 2006; Mörschel and Dutschmann, 2009).

Alternatively, our results suggest that the pontine-medullary feedback does not simply function as an “internal pulmonary feedback,” performing a redundant function and compensating for the potential loss of vagal input. The specific increase in the variability of *T*_*E*_ with the suppression pontine activity and the significant prolongation of *T*_*E*_ after vagotomy (Figure 6A) indicate that the pontine and pulmonary feedbacks differ in the control of *T*_*E*_. Indeed, our modeling results show that these control loops may complement each other in differential control of phase duration and breathing pattern variability. For example, an increase of *T*_*E*_ variability with pontine suppression, as seen in Figures 5B and 6A, may be the case during various breathing disorders, such as sleep apnea or ventilator weaning (Tobin et al., 2012). In this connection, the stability of *T*_*E*_ can be critically important and is primarily being controlled by the pons. Moreover, the Kölliker-Fuse area of the dorsolateral pons was explicitly identified to contribute to breathing disorders in a mouse model for a neurodevelopmental disease called Rett-syndrome (Stettner et al., 2007; Abdala et al., 2010).

Consistent with the many earlier and recent experimental data from cats and rats (Lumsden, 1923; Cohen, 1979; Wang et al., 1993; Jodkowski et al., 1994; Morrison et al., 1994; St. John, 1998), our simulations show that a strong pontine suppression (e.g., 75%) or its removal after vagotomy leads to apneusis, characterized by a significant increase of inspiratory duration and its variability (Figures 5C and 6A). The other specific characteristics of apneusis are a lack of post-inspiratory activity and a reduction of phrenic amplitude during inspiration (Cohen, 1979; Wang et al., 1993; Jodkowski et al., 1994; Morrison et al., 1994; Fung and St. John, 1995; St. John, 1998), which were reproduced in our simulations (Figure 5C).

Our understanding of interactions between individual components of complex systems is often insufficient to explain emergent properties of these systems. The present study elucidates the important role of two major feedback loops and interactions between them in regulation of the respiratory rate and breathing pattern allowing the brainstem respiratory network to maintain system's homeostasis and adjust breathing to various metabolic and physiologic demands.

### Conflict of interest statement

The authors declare that the research was conducted in the absence of any commercial or financial relationships that could be construed as a potential conflict of interest.

## Appendix

### Single neuron model

All neurons were modeled in the Hodgkin-Huxley style as single-compartment models:

(A1) $$C\cdot \frac{dV}{dt}=-I_{Na}-I_{NaP}-I_{K}-I_{CaL}-I_{K,\;Ca}-I_{L}-I_{SynE}-I_{SynI},$$

where *V* is the membrane potential, *C* is the membrane capacitance, and *t* is time. The terms in the right part of this equation represent ionic currents: *I*_*Na*_—fast sodium (with maximal conductance ${\bar{g}}_{Na}$); *I*_*NaP*_—persistent (slow inactivating) sodium (with maximal conductance ${\bar{g}}_{NaP}$); *I*_*K*_—delayed rectifier potassium (with maximal conductance ${\bar{g}}_{K}$); *I*_*CaL*_—high-voltage activated calcium (with maximal conductance ${\bar{g}}_{CaL}$); *I*_*K*, *Ca*_—calcium-dependent potassium (with maximal conductance ${\bar{g}}_{K,\;Ca}$), *I*_*L*_—leakage (with constant conductance *g*_*L*_); *I*_*SynE*_ (with conductance *g*_*SynE*_) and *I*_*SynI*_ (with conductance *g*_*SynI*_)—excitatory and inhibitory synaptic currents, respectively.

Currents are described as follows:

(A2) $$\begin{array}{l} \;I_{Na}={\bar{g}}_{Na}\;\cdot \;m_{Na}^{3}\;\cdot \;h_{Na}\;\cdot \;(V-E_{Na});\\ \;I_{NaP}={\bar{g}}_{NaP}\;\cdot \;m_{NaP}\;\cdot \;h_{NaP}\;\cdot \;(V-E_{Na});\\ \;I_{K}={\bar{g}}_{K}\cdot \;m_{K}^{4}\;\cdot \;(V-E_{K});\\ \;I_{CaL}={\bar{g}}_{CaL}\;\cdot \;m_{CaL}\;\cdot \;h_{CaL}\;\cdot \;(V-E_{Ca});\\ I_{K,\;Ca}={\bar{g}}_{K,\;Ca}\;\cdot \;m_{K,\;Ca}^{2}\;\cdot \;(V-E_{K});\\ \;I_{L}=g_{L}\;\cdot \;(V-E_{L});\\ \;I_{SynE}=g_{SynE}\;\cdot \;(V-E_{SynE});\\ \;I_{SynI}=g_{SynI}\;\cdot \;(V-E_{SynI}), \end{array}$$

where *E*_*Na*_, *E*_*K*_, *E*_*Ca*_, *E*_*L*_, *E*_*SynE*_, and *E*_*SynI*_ are the reversal potentials for the corresponding channels.

Variables *m*_*i*_ and *h*_*i*_ with indexes indicating ionic currents represent, respectively, the activation and inactivation variables of the corresponding ionic channels. Kinetics of activation and inactivation variables is described as follows:

(A3) $$\begin{array}{l} \tau_{mi}(V)\;\cdot \;\frac{d}{dt}m_{i}=m_{\infty i}(V)-m_{i};\\ \;\tau_{hi}(V)\;\cdot \;\frac{d}{dt}h_{i}=h_{\infty i}(V)-h_{i}. \end{array}$$

The expressions for steady state activation and inactivation variables and time constants are shown in Table A1. The value of maximal conductances for all neuron types are shown in Table A2.

**Table A1:** **Steady state activation and inactivation variables and time constants for different ionic channels**.

**Ionic channels**	*m*_∞_(*V*), *V* in mV;
	τ_*m*_(*V*), ms;
	*h*_∞_(*V*), *V* in mV;
	τ_*h*_(*V*), ms
Fast sodium Na	*m*_∞*Na*_ = 1/(1 + exp(−(*V* + 43.8)/6));
	τ_*mNa*_ = τ_*mNa* max_/ cosh ((*V* + 43.8)/14), τ_*mNa* max_ = 0.252;
	*h*_∞*Na*_ = 1/(1 + exp((*V* + 67.5)/10.8));
	τ_*hNa*_ = τ_*hNa* max_/ cosh ((*V* + 67.5)/12.8), τ_*hNa* max_ = 8.456
Persistent sodium NaP	*m*_∞*NaP*_ = 1/(1 + exp(−(*V* + 47.1)/3.1));
	τ_*mNaP*_ = τ_*mNaP* max_/ cosh ((*V* + 47.1)/6.2), τ_*mNaP* max_ = 1;
	*h*_∞*NaP*_ = 1/(1 + exp((*V* + 60)/9));
	τ_*hNaP*_ = τ_*hNaP* max_/ cosh (*V* + 60)/9), τ_*hNaP* max_ = 5000
Delayed rectifier potassium K	α_∞*K*_ = 0.01· (*V* + 44)/(1 − exp(−(*V* + 44)/5));
	β_∞*K*_ = 0.17· exp(−(*V* + 49)/40));
	*m*_∞*K*_ = α_∞*K*_/(α_∞*K*_ + β_∞*K*_).
	τ_*mK*_ = τ_*mK* max_/(α_∞*K*_ + β_∞*K*_), τ_*mK* max_ = 1
High-voltage activated calcium Ca_*L*_	*m*_∞*CaL*_ = 1/(1 + exp(−(*V* + 27.4)/5.7));
	τ_*mCaL*_ = 0.5;
	*h*_∞*CaL*_ = (1 + exp((*V* + 52.4)/5.2));
	τ_*hCaL*_ = 18
Calcium-dependent potassium K(Ca^2+^)	α_∞*K*, *Ca*_ = 1.25· 10^8^ · [*Ca*]^2^_*i*_, β_∞*K*, *Ca*_ = 2.5;
	m_∞*K*, *Ca*_ = α_∞*K*, *Ca*_/(α_∞*K*, *Ca*_ + β_∞*K*, *Ca*_).
	τ_*mK*, *Ca*_ = τ_*mK*, *Ca* max_ · 1000/(α_∞*K*, *Ca*_ + β_∞*K*, *Ca*_),
	τ_*mK* max_ = 0.7 − 1.0

**Table A2:** **Maximal conductances of ionic channels in different neuron types**.

**Neuron type**	**${\bar{g}}_{Na}$, nS**	**${\bar{g}}_{NaP}$, nS**	**${\bar{g}}_{K}$, nS**	**${\bar{g}}_{CaL}$, nS**	**${\bar{g}}_{K,\;Ca}$, nS**	***g*_*L*_, nS**
pre-I	170	5.0	180			2.5
post-I, post-Ie	400		250	0.1	6.0	6.0
aug-E	400		250	0.1	3.0	6.0
early-I(1)	400		250	0.1	3.5	6.0
early-I(2)	400		250	0.1	11.0	6.0
All others	400		250			6.0

The kinetics of intracellular calcium concentration *Ca* is described as follows (Rybak et al., 1997):

(A4) $$\frac{d}{dt}Ca=-k_{Ca}\;\cdot \;I_{CaL}\;\cdot \;(1-P_{B})+(Ca_{0}-Ca)/\tau_{Ca},$$

where the first term constitutes influx (with the coefficient *k*_*Ca*_) and buffering (with the probability *P*_*B*_), and the second term describes pump kinetics with resting level of calcium concentration *Ca*_0_ and time constant τ_*Ca*_.

(A5) $$P_{B}=B/(Ca+B+K),$$

where *B* is the total buffer concentration and *K* is the rate parameter.

The calcium reversal potential is considered a variable and is a function of *Ca*:

(A6) $$E_{Ca}=13.27\;\cdot \;\ln(4/Ca)\;(\text{at rest }Ca=Ca_{\text{o}}=5\times 10^{-5}\text{ mM and }E_{Ca}=150\text{ mV}).$$

The excitatory (*g*_*SynE*_) and inhibitory synaptic (*g*_*SynI*_) conductances are equal to zero at rest and may be activated (opened) by the excitatory or inhibitory inputs respectively:

(A7) $$\begin{array}{l} g_{SynEi}(t)={\bar{g}}_{E}\;\cdot \;F_{i}^{presyn}\;\cdot \;\sum_{j}S\{w_{ji}\}\;\cdot \;\sum_{t_{kj}<t}\exp\left(-\left(t-t_{kj}\right)/\tau_{SynE}\right)+{\bar{g}}_{Ed}\;\cdot \;\sum_{m}S\{w_{dmi}\}\;\cdot \;d_{mi};\\ g_{SynIi}(t)={\bar{g}}_{I}\;\cdot \;\sum_{j}S\left\{-w_{ji}\right\}\;\cdot \;\sum_{t_{kj}<t}\exp\left(-\left(t-t_{kj}\right)/\tau_{SynI}\right)+{\bar{g}}_{Id}\;\cdot \;\sum_{m}S\{-w_{dmi}\}\;\cdot \;d_{mi}, \end{array}$$

where the function *S*{*x*} = *x*, if *x* ≥ 0, and 0 if *x* < 0. In Equations (A7), each of the excitatory and inhibitory synaptic conductances has two terms. The first term describes the integrated effect of inputs from other neurons in the network (excitatory or inhibitory). The second term describes the integrated effect of inputs from external drives *d*_*mi*_. Each spike arriving to neuron *i* from neuron *j* at time *t*_*kj*_ increases the excitatory synaptic conductance by ${\bar{g}}_{E}\;\cdot \;w_{ji}$ if the synaptic weight *w*_*ji*_ > 0, or increases the inhibitory synaptic conductance by $-{\bar{g}}_{I}\;\cdot \;w_{ji}$ if the synaptic weight *w*_*ji*_ < 0. ${\bar{g}}_{E}$ and ${\bar{g}}_{I}$ are the parameters defining an increase in the excitatory or inhibitory synaptic conductance, respectively, produced by one arriving spike at |*w*_*ji*_| = 1. τ_*SynE*_ and τ_*SynE*_ are the decay time constants for the excitatory and inhibitory conductances respectively. In the second terms of Equation (A7), ${\bar{g}}_{Ed}$ and ${\bar{g}}_{Id}$ are the parameters defining the increase in the excitatory or inhibitory synaptic conductance, respectively, produced by external input drive *d*_*mi*_ = 1 with a synaptic weight of |*w*_*dmi*_| = 1. All drives were set to 1.

Presynaptic inhibition is simulated as an attenuator of excitatory synapses by means of a factor *F*^*presyn*^ ≤ 1. This factor is calculated according to the following equation:

(A8) $$F_{i}^{presyn}={\left(1+\sum_{j}S\left\{-w_{ji}^{\;p}\right\}\;\cdot \;\sum_{t_{kj}<t}\exp\left(-\left(t-t_{kj}\right)/\tau_{SynI}\right)\right)}^{-1},$$

where *w*^*p*^_*ji*_ ≤ 0 is the weight of presynaptic inhibitory connection that synapse *i* receives from neuron *j*. If a synapse *i* does not receive any presynaptic inhibition, then *w*^*p*^_*ji*_ = 0 for and hence for this synapse *F*^*presyn*^_*i*_ = 1.

The relative weights of synaptic connections (*w*_*ji*_, *w*^*p*^_*ji*_, and *w*_*dmi*_) are shown in Table A3.

**Table A3:** **Weights of synaptic connections in the network**.

**Target population (location)**	**Excitatory drive (weight of synaptic input from this drive) or source population (from single neuron)**
ramp-I (rVRG)	drive(Pons) (0.7);
	post-I (−1.0); aug-E(−0.15); pre-I /I (0.06); early-I(2) (−0.2);
	pontine I (0.2); Pe (0.115)
early-I(2) (rVRG)	drive(Pons) (2);
	post-I (−0.5);
	Pi (−0.15)
pre-I/I (pre-BötC)	drive(Pons) (0.03); drive(Raphe) (0.3); drive(RTN) (0.2);
	post-I (−0.1625); aug-E (−0.0275); pre-I /I (0.03);
	pontine I (0.2); Pe (0.025)
early-I(1) (pre-BötC)	drive(Pons) (0.75); drive(RTN) (2.03);
	post-I (−0.4); aug-E (−0.2); pre-I /I (0.04);
	pontine IEi (−0.15)
aug-E (BötC)	drive(Pons) (0.6); drive(RTN) (1.25);
	post-I (−0.09); early-I(1) (−0.135);
	Pi (−0.075)
post-I and post-Ie (BötC)	drive(Pons) (0.5);
	aug-E (−0.025); early-I(1) (−0.15);
	pontine IEe (0.35); pontine E (0.075); Pe (0.275)
pontine I (Pons)	drive(Pons) (0.25) (only to tonic subpopulation);
	ramp-I (0.025);
	Pi (−0.5^p^)
pontine IEe and IEi (Pons)	drive(Pons) (0.2) (only to tonic subpopulations);
	ramp-I (0.03); post-Ie (0.05);
	Pi (−0.5^p^)
pontine E (Pons)	drive(Pons) (0.3) (only to tonic subpopulations);
	post-Ie (0.05);
	Pi (−5.0^p^)
Pe and Pi (NTS)	PSRs (1.0)
Phrenic Nerve (PN)	ramp-I (0.065)
Lungs	PN (1.2)
PSRs	Lungs (3.0)

Values in brackets represent relative weights of synaptic inputs from the corresponding source populations;

p *presynaptic inhibition*.

The following neuronal and synaptic parameters were used:

$$\begin{array}{l} \;C=36\text{ pF};\;E_{Na}=55\text{ mV};\;E_{K}=-94\text{ mV};\;E_{SynE}=-10\text{ mV};\\ E_{SynI}=E_{Cl}=-75\text{ mV};\\ \;{\bar{g}}_{E}={\bar{g}}_{I}={\bar{g}}_{Ed}={\bar{g}}_{Id}=1.0\text{ nS};\;\tau_{SynE}=5\text{ ms};\;\tau_{SynI}=15\text{ ms};\\ \;Ca_{0}=5\times 10^{-5}\text{ mM};\;k_{Ca}=2\times 10^{-5}\text{ mM}/\text{C};\;\tau_{Ca}=250\text{ ms},\\ \;B=0.030\text{ mM};K=0.001\text{ mM}. \end{array}$$

### Modeling neural populations

Each functional type of neuron in the model was represented by a population of 50 neurons. Connections between the populations were established so that, if a population A was assigned to receive an excitatory or inhibitory input from a population B or external drive D, then each neuron of population A received the corresponding excitatory or inhibitory synaptic input from each neuron of population B or from drive D, respectively. The pontine I, IEi, IEe, and E population represent an exception: only half of each population (the tonic subpopulation) receives tonic drive (see in the section “Pontine Feedback Loop”). To provide heterogeneity of neurons within neural populations, the value of *E*_*L*_ was randomly assigned from normal distributions using average value ± SD. Leakage reversal potential for all neurons (except for the pre-I ones) was *E*_*L*_ = −60 ± 1.2 mV; for pre-I neurons *E*_*L*_ = −68 ± 1.36 mV.

### Modeling of lungs, PN, and PSR

The phrenic motoneuron population and phrenic nerve (*PN*) were not modeled. Integrated activity of the ramp-I population were considered as PN motor output. An increase in lung volume (lung inflation) *V* was modeled as a low-pass filter of PN activity:

(A9) $$\tau_{V}\;\cdot \;\frac{dV}{dt}=-V+w_{PN\to V}\;\cdot \;PN,$$

where τ_*V*_ = 100 ms is a lung time constant. The PSR output was considered proportional to the lung inflation *V*.
